# A live RSV vaccine with engineered thermostability is immunogenic in cotton rats despite high attenuation

**DOI:** 10.1038/ncomms13916

**Published:** 2016-12-21

**Authors:** Christopher C. Stobart, Christina A. Rostad, Zunlong Ke, Rebecca S. Dillard, Cheri M. Hampton, Joshua D. Strauss, Hong Yi, Anne L. Hotard, Jia Meng, Raymond J. Pickles, Kaori Sakamoto, Sujin Lee, Michael G. Currier, Syed M. Moin, Barney S. Graham, Marina S. Boukhvalova, Brian E. Gilbert, Jorge C. G. Blanco, Pedro A. Piedra, Elizabeth R. Wright, Martin L. Moore

**Affiliations:** 1 Department of Pediatrics, Emory University School of Medicine, Atlanta, Georgia 30322, USA; 2 Children's Healthcare of Atlanta, Atlanta, Georgia 30322, USA; 3 School of Biology, Georgia Institute of Technology, Atlanta, Georgia 30332, USA; 4 Robert P Apkarian Integrated Electron Microscopy Core, Emory University, Atlanta, Georgia 30322, USA; 5 Department of Microbiology and Immunology, University of North Carolina, Chapel Hill, North Carolina 27599, USA; 6 Department of Pathology, College of Veterinary Medicine, University of Georgia, Athens, Georgia 30602, USA; 7 Vaccine Research Center, National Institute of Allergy and Infectious Diseases, National Institutes of Health, Bethesda, Maryland 20852, USA; 8 Sigmovir Biosystems Inc., Rockville, Maryland 20850, USA; 9 Department of Molecular Virology and Microbiology, Baylor College of Medicine, Houston, Texas 77030, USA; 10 Department of Pediatrics, Baylor College of Medicine, Houston, Texas 77030, USA

## Abstract

Respiratory syncytial virus (RSV) is a leading cause of infant hospitalization and there remains no pediatric vaccine. RSV live-attenuated vaccines (LAVs) have a history of safe testing in infants; however, achieving an effective balance of attenuation and immunogenicity has proven challenging. Here we seek to engineer an RSV LAV with enhanced immunogenicity. Genetic mapping identifies strain line 19 fusion (F) protein residues that correlate with pre-fusion antigen maintenance by ELISA and thermal stability of infectivity in live RSV. We generate a LAV candidate named OE4 which expresses line 19F and is attenuated by codon-deoptimization of non-structural (NS1 and NS2) genes, deletion of the small hydrophobic (SH) gene, codon-deoptimization of the attachment (G) gene and ablation of the secreted form of G. OE4 (RSV-A2-dNS1-dNS2-ΔSH-dG_m_-Gs_null_-line19F) exhibits elevated pre-fusion antigen levels, thermal stability, immunogenicity, and efficacy despite heavy attenuation in the upper and lower airways of cotton rats.

## Abstract (web summary)

Development of a safe and efficacious vaccine for respiratory syncytial virus (RSV) has been challenging. Here the authors generate a live-attenuated RSV vaccine that shows increased thermostability and immunogenicity, and protects cotton rats from RSV challenge.

In the 1960s, a formalin-inactivated RSV (FI-RSV) vaccine primed for enhanced illness in infants on natural infection^1^. This phenomenon was replicable in animal models and considered dependent on RSV naive status^2^. Subsequent studies using subunit-based vaccines also primed for immunopathology in animals^3^,^4^. These early RSV vaccines encouraged development of LAVs, which do not prime for enhanced disease in animals or seronegative infants^2^,^5^. However, development of pediatric RSV LAV strains with sufficient attenuation and immunogenicity has been difficult^6^. To address these dual challenges, newer RSV LAVs have incorporated genetic modifications rationally designed to retain or enhance immunogenicity compared with wild-type virus^7^,^8^,^9^ because natural infection may be suboptimally immunogenic for LAVs derived by classic attenuation methods.

Recent elucidation of the structure of the pre-fusion conformation of RSV F protein (pre-F^10^) and discovery of its importance as a natural immunogen^11^ has had implications for RSV vaccine development. The high capacity of pre-F to elicit neutralizing antibody titres has been demonstrated in multiple vaccine platforms, including purified proteins^12^,^13^,^14^, virus-like particles^15^, and recombinant parainfluenza viruses^16^. Use of pre-F in passive immunization, either by anti-pre-F monoclonal antibody (mAb) prophylaxis or by boosting RSV neutralizing antibody (nAb) titres in pregnant mothers with pre-F protein-based vaccines, holds promise for reducing RSV disease in the youngest infants^14^. Nevertheless, active immunization of infants with a replicating RSV vaccine could potentially have a large child health benefit if protection spanned beyond the persistence of passively acquired maternal Ab. Since natural RSV infection induces anti-pre-F nAb^11^, we hypothesized that RSV with enhanced pre-F expression would have increased LAV immunogenicity.

Here we first identified a chimeric RSV strain A2-line19F with enhanced pre-fusion antigen levels, thermostability and immunogenicity compared with parental strain A2. We then incorporated line19F into an RSV LAV candidate ‘OE4' with the genotype RSV-A2-dNS1- dNS2-ΔSH-dG_m_-Gs_null_-line19F. We found that OE4 exhibited elevated pre-fusion antigen levels, thermal stability, immunogenicity, and efficacy despite heavy attenuation in the upper and lower airways of cotton rats.

## Results

### Pre-fusion F ELISAs

**Figure 1:**
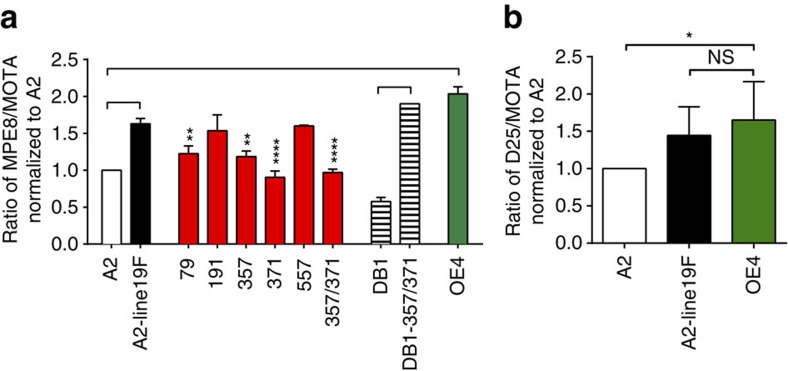
MPE8 and D25 ELISAs. (**a**) Ratio of direct ELISA using MPE8, a pre-F-specific mAb, to direct ELISA using motavizumab, a total F mAb. Values are normalized to strain A2. For A2-line19F mutants, the asterisks show significant differences compared with A2-line19F. (**b**) Ratio of direct ELISA using D25, another pre-F-specific mAb, to direct ELISA using motavizumab. All graphs represent the means+s.d.'s of at least two experimental replicates, and data were analysed by one-way ANOVA. When significant, *P* values are shown as a bracket between groups (*P*<0.0005) or by asterisk when compared with A2-line19F (*P<0.05; **P<0.005; ***P<0.0005).

Metastable pre-F undergoes a dynamic transition to form a thermodynamically stable six-helix post-fusion bundle that facilitates viral and host membrane fusion^10^,^14^. Since both pre-F and post-F are present on RSV virions in prepared virus stocks^17^,^18^, we evaluated the relative amount of pre-F antigen in RSV stocks using an ELISA-based approach to compare MPE8 with motavizumab antibody binding. MPE8 is a human monoclonal antibody that preferentially binds to two highly conserved anti-parallel β-strands on pre-F, which are rearranged in the post-fusion conformation to render them less accessible to antibody binding^19^. Motavizumab, in contrast, stably binds to both pre- and post-fusion F. We found that strain A2-line19F, which expresses the F protein of strain line 19 in the background of the prototypical A2 strain^20^,^21^, exhibited significantly higher relative binding to MPE8 than did strain A2 (Fig. 1a). We confirmed this finding using the human monoclonal antibody D25, which binds to a distinct antigenic site on pre-F (antigenic site Ø)^10^ with even greater specificity than MPE8 (ref. 22). We found that A2-line19F exhibited higher relative binding to D25 than A2, which was similar in magnitude and correlated with MPE8 binding (Fig. 1b).

Five unique amino-acid residues distinguish line 19F from A2 F: M79, R191, K357, Y371 and I557 (Supplementary Fig. 1)^20^. We generated A2-line19F mutants by substituting A2 residues in place of the unique line 19F residues^20^. To determine the effects of these residues on pre-F antigen levels in virus stocks, we performed MPE8 and motavizumab ELISAs on the recombinant A2-line19F mutant viruses. We found that residues M79, K357 and Y371 contributed to line 19 F pre-F antigen levels (Fig. 1a). These results were consistent with previous data showing that the K357/Y371 residues together impeded A2-line19F fusion activity *in vitro*^20^.

### Thermal stability assays

**Figure 2:**
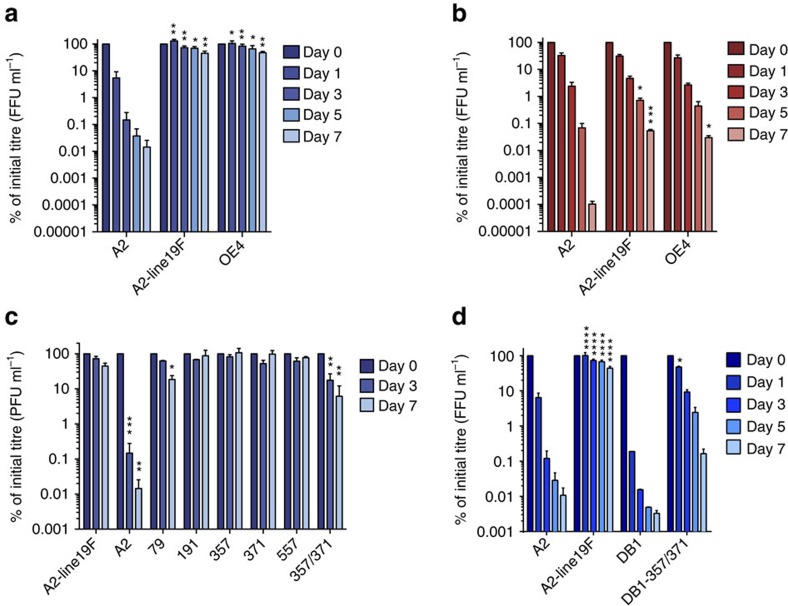
Thermal stability assays. Thermal inactivation was carried out either by incubation of virus at 4 °C (**a**,**c**,**d**) or 37 °C (**b**). The viruses in **c** labelled 79, 191, 357, 371, 557 and 357/371 represent A2-line19F containing substitutions at these indicated positions with A2 residues. The virus in **c** labelled DB1-357/371 represents DB1 with substitutions of line 19F residues at positions 357 and 371. Viruses were harvested at the indicated time points and titrated by FFU or PFU assays. All graphs represent the means+s.d.'s of at least two experimental replicates combined, and data were analysed by two-way ANOVA (*P<0.05; **P<0.005; ***P<0.0005; ****P<0.00005).

We next evaluated the thermal stability of A2-line19F compared with A2. RSV is known to be a heat-labile virus, and elevated temperatures can trigger the transition from pre- to post-fusion F^23^. We therefore hypothesized that RSV with enhanced pre-F levels would be more resistant to temperature inactivation. We analysed thermostability at 4 and 37 °C because thermostability at 4 °C may have implications for retention of vaccine potency in cold chain, whereas thermostability at 37 °C has more relevance for physiological conditions. We found that RSV A2-line19F infectivity was more thermostable over time than A2 at both temperatures (Fig. 2a,b), a phenotype that was mediated in part by the residues K357 and Y371 (Fig. 2c). We then introduced K357 and Y371 into the F of a genetically divergent vaccine strain DB1, which expresses a consensus F gene of the antigenic subgroup B ‘Buenos Aires' (BAF) clade. We previously described the generation of DB1, which also contains codon-deoptimized non-structural protein genes and deleted SH gene, with a genotype RSV-A2-dNS1-dNS2-ΔSH-BAF^9^. DB1 expressed low levels of pre-F antigen and was thermally unstable; however, incorporation of the K357 and Y371 residues to generate DB1–357/371 enhanced MPE8 binding (Fig. 1a) and partially restored thermal stability (Fig. 2d). These data demonstrated that residues 357 and 371 modulated not only MPE8 binding, a correlate of pre-F antigen levels, but also viral resistance to thermal inactivation in viral stocks.

### Generation of RSV live-attenuated vaccine OE4

**Figure 3:**
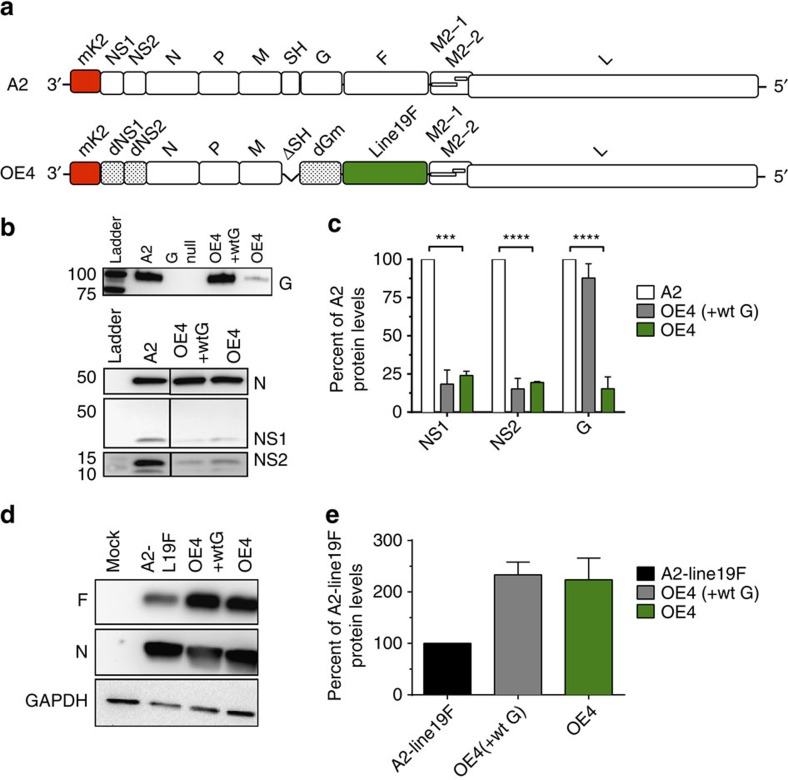
Design of live-attenuated vaccine OE4 and expression of viral proteins. (**a**) Schematic of RSV LAV OE4 genome including codon deoptimization of the NS1, NS2 and G genes, deletion of the SH gene, and incorporation of the line 19F gene. (**b**) Western blotting of Vero cells infected with A2 (white), OE4 (green) or OE4 expressing wild-type G (OE4-wtG, grey) for NS1, NS2, N and G. An A2-G_null_ mutant was included as a control. (**c**) Western densitometry analyses were normalized to A2 expression levels. (**d**) Western blotting of Vero cells infected with mock, A2-line19F (white), OE4-wtG (grey) or OE4 (green) for F, N and GAPDH. (**e**) Densitometry results were normalized to A2 expression levels. Densitometry results represent the means+s.d.'s of at least two experimental replicates and representative blots are shown. Statistical analyses were performed by one-way ANOVA (***P<0.0005; ****P<0.00005). d, codon-deoptimized; F, fusion protein; G, attachment glycoprotein; L, large polymerase; M, matrix; mK2, monomeric Katushka2; N, nucleoprotein; NS1/NS2, nonstructural proteins 1 and 2; P, phosphoprotein; SH, small hydrophobic protein.

We next generated a novel RSV LAV called OE4, by incorporating line 19F into a multi-component vaccine designed to achieve attenuation, improved immunogenicity and genetic stability. We previously codon-deoptimized the NS1 and NS2 genes, which encode two nonstructural proteins of RSV that suppress host innate immunity by targeting interferon pathways and suppressing apoptosis^24^,^25^. Codon deoptimization of NS1 and NS2 genes was genetically stable and reduced NS1 and NS2 protein expression, resulting in virus attenuation with slightly enhanced immunogenicity in mice^8^. We subsequently deleted the small hydrophobic (SH) protein gene with the goal of increasing the transcription of downstream viral genes, including F, by altering their proximity to the viral leader^26^. The deletion of SH is also mildly attenuating in mice and chimpanzees, but conferred no apparent attenuation in a vaccine candidate in children^26^,^27^,^28^. Last, we codon-deoptimized the RSV attachment (G) glycoprotein gene and ablated the secreted form of G by a point mutation. RSV expresses a membrane-bound form (G_m_) and a secreted form (G_s_) of G, which are not required for viral replication in immortalized cell lines^29^,^30^,^31^,^32^. RSV G is capable of eliciting protective neutralizing antibodies^33^. However, G is less conserved than F and suppresses the innate immune response through chemokine mimicry^34^,^35^. G_s_ functions as an antigen decoy and can alter dendritic cell signalling and activation through interactions with C-type lectins^36^,^37^. The resulting genotype of the OE4 vaccine candidate was RSV-A2-dNS1-dNS2-ΔSH-dG_m_-Gs_null_-line19F (Fig. 3a). Using western blotting, we demonstrated that OE4 had decreased expression levels of NS1, NS2 and G as expected compared with parental A2 (Fig. 3b,c). We additionally found that OE4 had higher levels of F expression than A2-line19F, likely attributable to the deletion of SH (Fig. 3d,e).

### Analysis of OE4 surface glycoproteins

We analysed the MPE8 and D25 binding of OE4 and measured vaccine thermal stability at 4 and 37 °C. Similar to A2-line19F, OE4 exhibited high relative pre-F antigen levels by antibody binding (Fig. 1a,b) and thermal stability (Fig. 2a,b) consistent with its expression of the line 19F protein. We further explored this relationship by quantifying pre-F stability as measured by MPE8 binding of OE4 and A2 from virus stocks incubated at 4 °C over time. Relative pre-F antigen levels declined in both viruses over a period of 8 days (Supplementary Fig. 2a). Therefore, the kinetics of thermal stability of A2 and OE4 infectivity did not correlate with the decay of pre-F antigen levels. However, OE4 maintained greater than twice the levels of pre-F antigen levels at each time point compared with A2 (Supplementary Fig. 2b), and a minimal threshold of pre-F may be sufficient to maintain infectivity.

**Figure 4:**
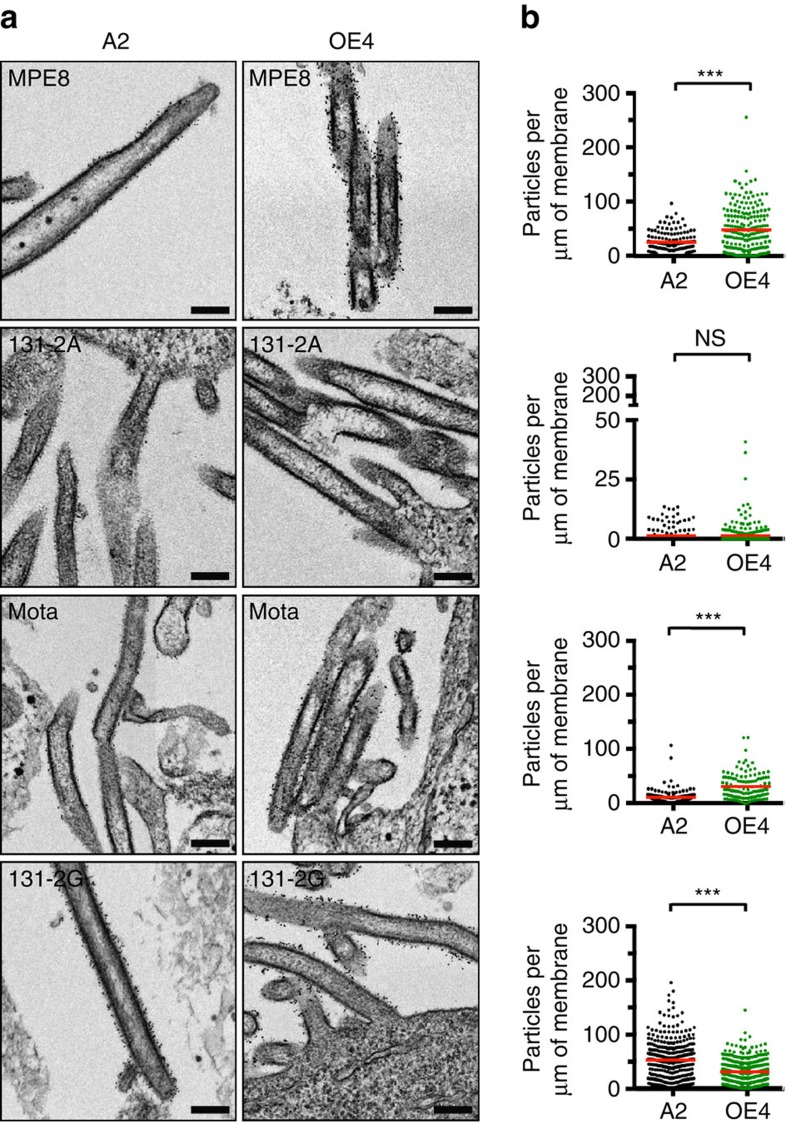
Immunogold labelling of RSV surface glycoproteins F and G. (**a**) Representative TEM images of BEAS-2B cells infected at an MOI of 10 with A2 (black) or OE4 (green) and labelled with MPE8 (pre-F mAb), 131-2A (post-F mAb), Motavizumab (total F mAb) or 131-2G (G mAb) and probed with gold-labelled secondary antibodies. (**b**) Quantification of the amount of immunogold particles per measured membrane length per virion. For each labeling condition, more than 100 virions (graph data points) were evaluated for each virus. The red lines represent the mean particle densities along the membrane for each condition. Significant differences are indicated by ***P<0.0005 determined by *t*-test with Welch's correction. Scale bars represent 200 nm.

In order to assess the overall structure of the virions and glycoprotein incorporation into RSV A2 and OE4, we then performed thin-section transmission electron microscopy (TEM), native immuno-TEM, and cryo-electron tomography (cryo-ET) of viruses budded from BEAS-2B cells, an immortalized human bronchial epithelial cell line. In all cases, virus-infected cells and released virions were analysed following minimal sample processing to maximize preservation of the native structure of the virions. First, native immunogold labelling combined with thin-section TEM was performed using mAbs that preferentially bound pre-F (MPE8), post-F (131-2A), total F (motavizumab) or G (131-2G) (Fig. 4a)^38^. The density of gold particles per membrane length was quantified for each virus and immunolabel (Fig. 4b)^39^. OE4 virus particles exhibited a greater density of incorporated pre-F and total F than A2, potentially due to the deletion of SH. There was no significant difference in the amount of post-F detected on the surfaces of A2 and OE4 particles. G protein density on OE4 particles was significantly reduced, as was expected in the setting of codon-deoptimization of the G gene.

**Figure 5:**
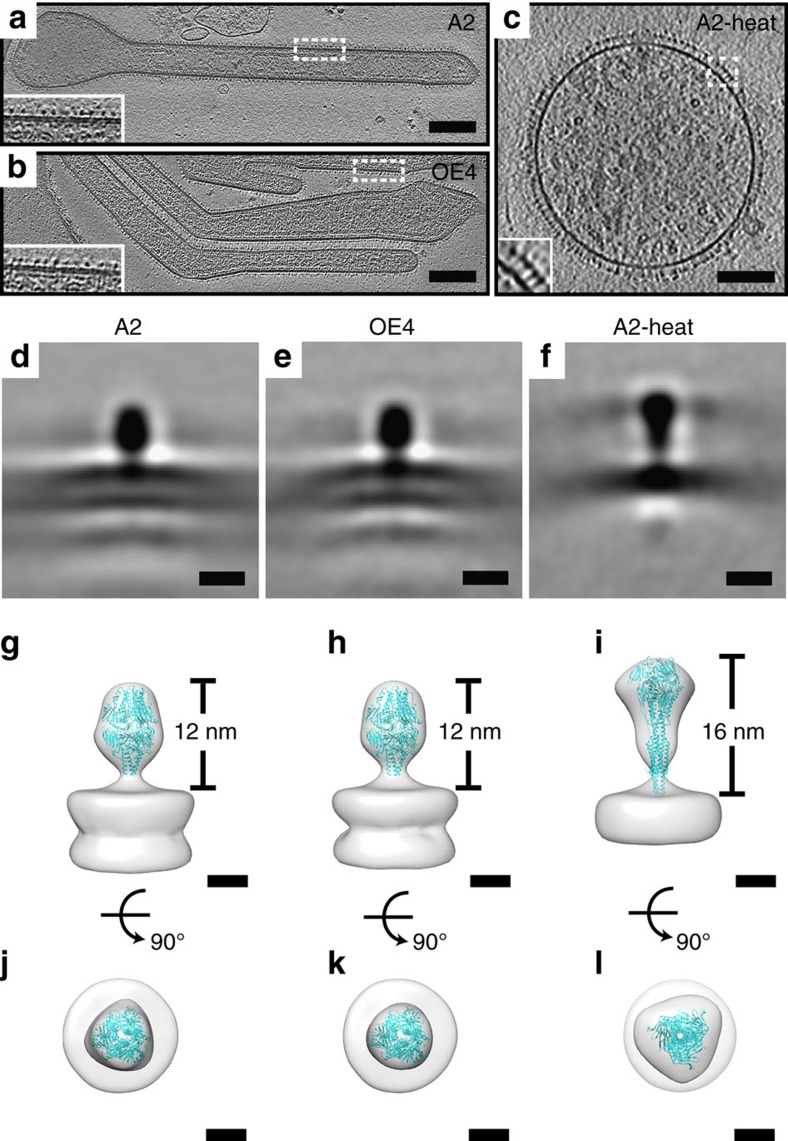
Cryo-electron tomography of RSV virions and subvolume averaging of the F glycoprotein. (**a**–**c**) Tomographic slices (6.14 nm) of A2, OE4 and A2-heat (55 °C for 30 min) virions showing overall virus structure and the organization of surface glycoproteins (insets). Inset in OE4 is rotated 180°. Scale bars are 200 nm for A2 and OE4, and 100 nm for A2-heat. (**d**–**l**) Subvolume averages and modelling of RSV F structures in pre- and post-fusion conformations. Central slices (6.14 Å in thickness) of the averaged structures lowpass filtered to 40 Å for A2 (**d**), OE4 (**e**) and A2-heat (**f**). Quasi-atomic models generated by fitting the RSV pre-fusion F (PDB ID 4JHW) and RSV post-fusion F (PDB ID 3RRT) crystal structures into the subvolume averages, with side views (**g**–**i**) and top views (**j**–**l**) for A2 (**g**,**j**), OE4 (**h**,**k**) and A2-heat (**i**,**l**). Note the height difference between the ectodomain of A2/OE4 and A2-heat. The measurements were made from the top of the membrane to the top of the head domain. Scale bars, 10 nm (**d**–**f**); 5 nm (**g**–**l**).

When visualized by cryo-ET, A2 and OE4 virus particles were morphologically similar and formed filaments with abundant glycoprotein spikes on the surface, matrix protein lining the inside of the viral membrane, and ribonucleoprotein complex in the interior of the virions (Fig. 5a–c)^17^,^18^,^38^. To further investigate the conformations of RSV F on the surfaces of A2 and OE4 virions in their native states, we then calculated subvolume averages of F structures from the cryo-ET data. These studies demonstrated that the majority of F proteins on both viruses in their native states immediately after budding was in the pre-F conformation (Fig. 5). The application of heat (55 °C for 30 min) triggered the conformational change from pre- to post-F, providing direct evidence of the relationship between temperature and pre-F stability (Fig. 5c,f,i,j).

### Characterization of OE4 in cell culture and primary cells

**Figure 6:**
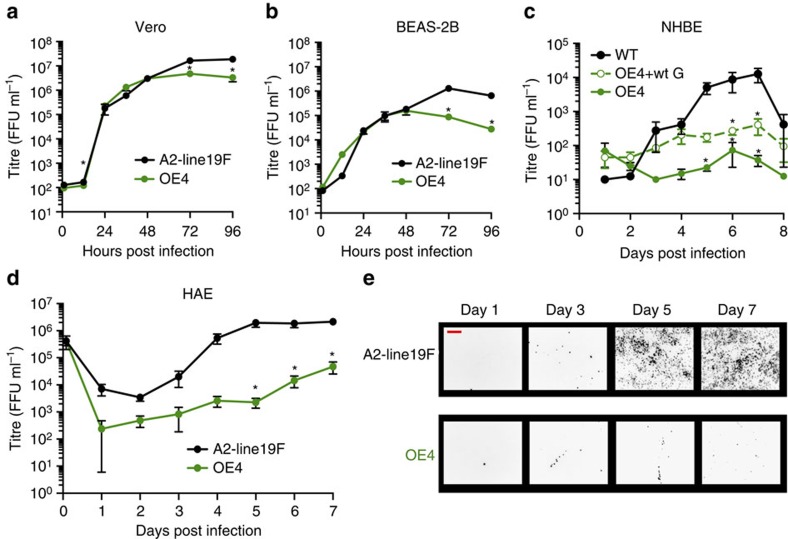
OE4 replication in immortalized and primary cell cultures. Vero (**a**), BEAS-2B (**b**), primary normal human bronchial epithelial cells differentiated at air–liquid interface (NHBE) (**c**), primary human tracheobronchial airway cells differentiated at air–liquid interface (HAE) (**d**) were infected with A2-line19F (black), OE4 (green) and in HAE, OE4+wtG (green dash) at MOI=0.01 (Vero and BEAS-2B), MOI=2.6 (NHBE), or MOI=6.7 (HAE). Samples were titrated by fluorescent focus unit (FFU) assays on Vero cells. (**e**) Representative images of infected HAE cultures. Scale bar represents 200 μm. Graphs depict the means±s.e.s of the means combined from three experiments (Vero and BEAS-2B), from two donors in duplicate (NHBE), or from six cultures from a single donor per virus (HAE). When significant, *P* values are shown relative to A2-line19F (*P<0.05; by two-way ANOVA).

We next characterized the OE4 vaccine candidate *in vitro* by measuring attenuation levels in immortalized cells and in primary human airway epithelial cells (Fig. 6). In Vero cells, which were used for virus stock generation, OE4 grew to titres slightly below the parental unattenuated A2-line19F (Fig. 6a). OE4 was more attenuated relative to wild type in BEAS-2B cells (Fig. 6b). We then evaluated OE4 growth in primary human airway epithelial cells, which are an established system for approximating RSV LAV attenuation in seronegative children^40^. We implemented two models, NHBE-ALI and HAE-ALI, and found that OE4 was significantly attenuated in both models (Fig. 6c,d) and exhibited deficiency in spreading through the cultures (Fig. 6e). The codon deoptimization of G in OE4 contributed significantly to the level of attenuation compared with OE4 expressing wild-type G (OE4+wtG) in NHBE-ALI (Fig. 6c), likely due to the previously described attachment role of G in primary cells^30^.

### Characterization of OE4 in BALB/c mice

**Figure 7:**
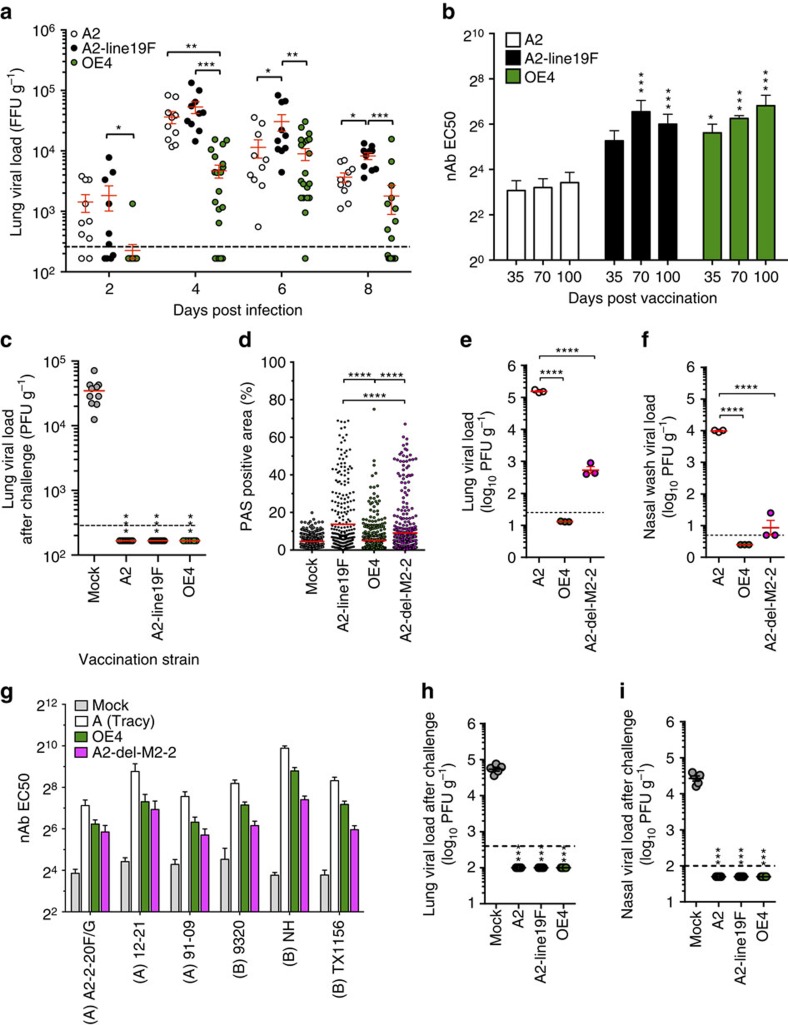
Attenuation and efficacy of OE4 in mice and cotton rats. (**a**) Lung viral loads were determined in mice inoculated i.n. with 10^6^ FFU of A2, A2-line19F or OE4 at the indicated time points. (**b**) Serum nAb titres were measured in mice inoculated with 10^6^ FFU of A2, A2-line19F or OE4. (**c**) Mice were inoculated with 10^6^ FFU of A2, A2-line19F or OE4 then challenged with 10^5^ PFU of A2-line19F on day 102. Lung viral loads were determined day 4 post challenge. (**a**–**c**) Graphs represent combined data from two experiments of 5–10 mice per group. (**d**) Mice (five per group) were inoculated with mock, A2-line19F, OE4 or A2-del-M2-2, and lungs were harvested 8 days post inoculation for histological quantification of airway mucin expression. Each dot represents an airway, and graph shows >300 airways per group in one of two experiments with similar results. (**e**,**f**) Viral load on day 4 in cotton rat lung homogenates (*n*=3) (**e**) and nasal washes (*n*=3) (**f**) following i.n. inoculation with 10^5^ FFU of A2, OE4 or A2-del-M2-2. (**g**) Cotton rats (six per group) were inoculated with mock, RSV A(Tracy), OE4 or A2-del-M2-2, and serum nAb titres against representative RSV strains were determined on day 42 post inoculation using pooled sera. EC50 was calculated by non-linear regression, and data represent EC50+upper limit of the 95% confidence interval. (**h**,**i**) Cotton rats (five per group) were inoculated with mock, A2, or OE4, challenged on day 42 with 10^6^ FFU of RSV A2-line19F, and viral loads on day 46 were measured in nasal washes (**i**) and lung lavages (**h**). *P<0.05; **P<0.005; ***P<0.0005; ****P<0.00005 by one-way (**c**–**f**,**h**,**i**) or two-way (**a**,**b**) ANOVA.

To measure relative levels of attenuation *in vivo*, we inoculated mice intranasally (i.n.) and measured lung viral loads on days 2, 4, 6 and 8 post infection. We found that OE4 was moderately attenuated compared with A2 and A2-line19F in this model (Fig. 7a). We compared lung viral loads of mice inoculated with OE4 with and without the mKate2 gene and found that reporter had no effect on viral attenuation (Supplementary Fig. 3), consistent with previously published results^41^. We sequenced the NS1, NS2, G and F genes of OE4 from virus recovered from mouse lungs on day 6 post infection, and there were no mutations in these genes. To analyse immunogenicity, we then vaccinated mice and measured nAb titres on days 35, 70 and 100 and found that OE4 elicited nAb titres equivalent to A2-line19F and higher than A2 at each time point post infection (Fig. 7b). Following i.n. inoculation, the mice were then challenged on day 102 with A2-line19F, and the OE4-vaccinated mice were completely protected against the challenge (Fig. 7c). We next measured OE4 mucus production in the lungs of mice. We previously demonstrated that A2-line19F induces increased airway mucin expression, a measure of pathogenicity in this model^20^,^21^. However, OE4, which also expresses line 19F, induced lower levels of airway mucin expression than A2-line19F in mice (Fig. 7d and Supplementary Fig. 4), indicating that the attenuating genetic modifications in OE4 modulated the mucogenic phenotype. Subsequently, we compared OE4-induced mucin expression with a reconstituted RSV mutant containing a deletion of M2-2 (A2-del-M2-2), the primary genetic modification of a clinically advanced LAV candidate^7^. The deletion of M2-2 results in reduced viral replication and elevated transcription of downstream RSV genes (for example, NS1, NS2, SH, G, F and so on), which represents a different attenuation strategy than OE4. It should be noted that our reconstituted A2-del-M2-2 is not identical to MEDI-ΔM2-2 due to minor genetic differences between the A2 backbones. Because the deletion of M2-2 results in increased levels of non-essential virulence proteins, we hypothesized that A2-del-M2-2 would be mucogenic in mice. Compared with OE4, we found that A2-del-M2-2 induced significantly more airway mucin expression (Fig. 7d and Supplementary Fig. 4).

### Characterization of OE4 in cotton rats

OE4 attenuation and immunogenicity were next evaluated in cotton rats, a more permissive model of RSV infection. In cotton rats, OE4 was highly attenuated in the upper and lower respiratory tracts, and more attenuated than A2-del-M2-2 (Fig. 7e,f). Despite significant attenuation, OE4 induced relatively high levels of serum nAb against a panel of RSV strains representing RSV diversity (Fig. 7g). OE4-vaccinated cotton rats were completely protected against RSV challenge, not only in lungs (Fig. 7h) but also in the upper respiratory tracts (Fig. 7i). Thus, OE4 established effective mucosal immunity despite being highly attenuated in cotton rats.

**Figure 8:**
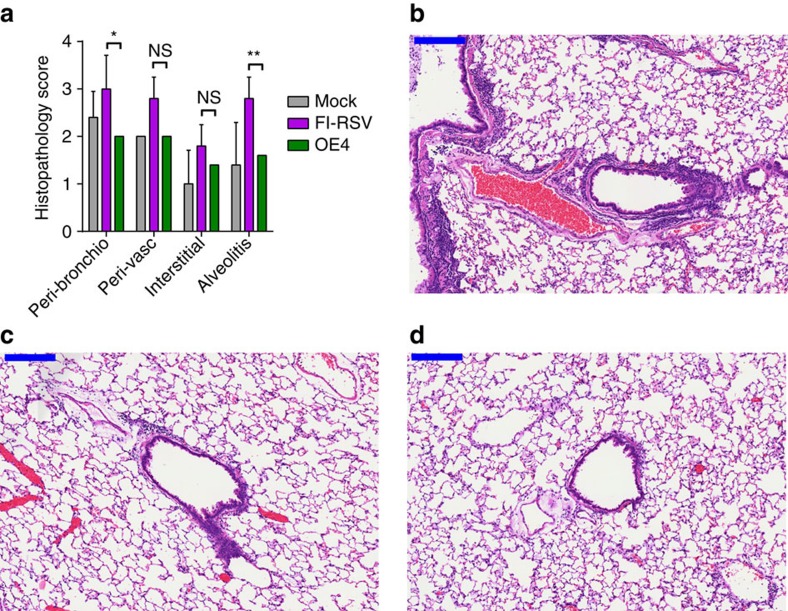
Histopathology following RSV challenge in cotton rats. To evaluate for vaccine attributable enhanced disease post challenge, groups of five cotton rats were inoculated intramuscularly with FI-RSV or intranasally with either mock or OE4. Animals vaccinated with FI-RSV also received a boost on day 21 p.i. All animals were challenged with A2-line19F on day 42 p.i., lungs were harvested 4 days later and histopathology scores were performed (**a**). Representative haematoxylin and eosin stains for FI-RSV (**b**), mock (**c**) and OE4 (**d**) vaccinated rats are shown. Scale bars represent 200 μm. Data are represented as mean+s.d. *P<0.05, **P<0.005 by two-way ANOVA.

Last, a primary concern highlighted by the failure of the FI-RSV vaccine candidate is the potential for vaccine-enhanced priming for disease on natural RSV infection. Although RSV LAV candidates have not been shown to cause enhanced illness, we evaluated whether the novel vaccination strategy employed by OE4 would prime for enhanced disease upon challenge in cotton rats. Results demonstrated that RSV challenge did not cause enhanced histopathology following infection with OE4 compared with mock (Fig. 8). In contrast, FI-RSV did result in enhanced disease associated with elevated peribronchiolar infiltration and alveolitis.

## Discussion

We identified a chimeric RSV strain A2-line19F that had increased relative MPE8 binding, increased thermal stability in viral stocks and increased immunogenicity *in vivo* compared with parental strain A2. A2-line19F differs from A2 by only five unique residues within the F protein. Incorporation of two of these residues (357/371) into a heterologous vaccine strain DB1 conferred increased relative MPE8 binding and increased thermal stability at 4 °C. To exploit these properties in an RSV LAV, we incorporated the line 19F protein into a rationally designed vaccine candidate OE4 with the genotype RSV-A2-dNS1-dNS2-ΔSH-dG_m_-G_Snull_-line19F. Like A2-line19F, OE4 had increased relative MPE8 and D25 binding and increased thermal stability compared with RSV A2. OE4 was also immunogenic and highly efficacious in BALB/c mice and cotton rats, despite significant levels of attenuation *in vitro* and *in vivo*. The mutations incorporated into OE4 were genetically stable in virus recovered from BALB/c mice. Furthermore, lung histopathologic staining demonstrated that OE4 was not mucogenic in mice, nor did it cause enhanced histopathology following RSV challenge in cotton rats.

One inherent limitation of our study is that neither mice nor cotton rats fully recapitulate RSV disease in humans. In our study, for example, we observed a difference in the attenuation levels of OE4 in our two animal models. Whereas OE4 was highly attenuated in cotton rats and in human primary airway epithelial cells, it was less attenuated in BALB/c mice. We also found that OE4 was more immunogenic in BALB/c mice than in cotton rats. We suspect these discrepancies were attributable to strain-specific differences in the attachment and infectivity of the line 19F protein and the differential effects of codon-deoptimized G protein in cotton rats compared with mice. For example, Teng *et al*. demonstrated that deletion of G from an RSV clinical stain was completely attenuating in cotton rats^32^, whereas Widjojoatmodjo *et al*.^42^ found that RSV-ΔG was only moderately attenuated in mice. Nevertheless, OE4 was significantly attenuated in both animal models and was capable of inducing protective neutralizing antibodies.

A second limitation of our study relates to the utilization of MPE8 and D25 ELISAs to quantify pre-F antigen levels in viral stocks. Both MPE8 and D25 are monoclonal antibodies that preferentially bind to the pre-fusion conformation of F; however, the conformational specificities of these two antibodies have not been fully validated. MPE8, in particular, competes with palivizumab^19^ and binds only 10–20 times better to pre- than post-fusion F^22^. D25, in contrast, binds at the apex of the pre-fusion trimer at the antigenic site Ø which undergoes marked structural rearrangement upon transition to post-F (ref. 10). Thus, D25 binds with even greater specificity to pre- than post-F (100-fold); ref. 22. Nevertheless, limited cross-reactivity with post-F has been observed, and a monomeric form of F has also been identified which retains pre-fusion-specific epitopes^13^,^43^. Despite these limitations, both MPE8 and D25 demonstrate relatively high pre-F specificity, and generated consistent results among the viruses analysed in this study.

Native immunogold labelling combined with thin-section TEM also demonstrated increased pre-F and total F on the surface of OE4 compared with A2. We suspect the increased incorporation of total F into OE4 was attributable to the deletion of the SH gene, which shifted the F gene towards the viral promoter. The vast majority of F in both OE4 and A2 was in the pre-fusion conformation, likely because the virions were maintained in their native states and not subjected to viral harvesting and stock preparation. Subvolume averaging of the F structures confirmed that the majority of F was in the pre-fusion conformation. However, application of heat to A2 triggered the conformational change to post-F, clearly demonstrating a relationship between temperature and pre-F stability. Although these results demonstrate that heat triggers the transition from pre- to post-fusion F, the relationship between temperature and pre-F stability remains incompletely defined. Similarly, the favourable immunogenicity to attenuation profile of OE4 is likely multifactorial and cannot be attributed specifically to the expression of pre-fusion F or to thermostability.

In conclusion, we identified key molecular determinant positions of RSV line 19F which were associated with both thermal stability and the availability of the pre-F antigen. Genetically modifying these residues to thermally stabilize and boost immunogenicity of RSV LAVs represents a promising new approach to next-generation RSV vaccine design. Using reverse genetics, we rationally designed a novel RSV LAV OE4 that incorporated line 19F into the genotype RSV-A2-dNS1-dNS2-ΔSH-dG_m_-G_Snull_-line19F. In addition to being thermally and genetically stable, OE4 was also highly immunogenic and efficacious despite significant attenuation *in vitro* and *in vivo*. These data demonstrate that we fundamentally altered RSV immunogenicity and generated a promising LAV candidate that merits further investigation.

## Methods

### Cells and animals

HEp-2 (ATCC CCL-23) and Vero (ATCC CCL-81) were cultured in minimal essential medium (MEM) containing 10% fetal bovine serum (FBS) and 1 μg ml^−1^ penicillin, streptomycin and amphotericin B (PSA)^8^. BSR-T7/5 (a gift from Ursula Buchholz, National Institutes of Health, Bethesda, MD) were cultured in Glasgow's minimal essential medium (GMEM) containing 10% FBS and 1 μg ml^−1^ PSA supplemented with 1 mg ml^−1^ Geneticin with every other passage^8^. BEAS-2B cells (ATCC CRL-9609) were cultured in RMPI containing 10% FBA and 1 μg ml^−1^ PSA^44^. The cell lines were not authenticated, and they were negative for mycoplasma using the LookOut Mycoplasma detection kit (Sigma). Normal human bronchial epithelial (NHBE) cells were purchased from Lonza and differentiated 4–6 weeks at air–liquid interface (ALI) as described^8^. Prior to infection, NHBE-ALI cultures exhibited trans-epithelial resistance. Human airway epithelial (HAE) cells from airway specimens of patients without defined lung disease were isolated by the University of North Carolina (UNC) Marsico Lung Institute Tissue Culture Core^45^. Patients provided informed consent under UNC at Chapel Hill Institutional Review Board-approved protocols from the National Disease Research Interchange (NDRI, Philadelphia, PA). Primary cells were cultured initially in a cell culture-treated flask before being seeded at a density of 3 × 10^5^ cells per Transwell disk. Similar to NHBE cells, HAE cells were cultured at ALI for 4–6 weeks forming differentiated polarized cultures^45^.

Six- to eight-week-old female BALB/c mice (The Jackson Laboratory or Charles River) were maintained under pathogen-free conditions until the time of use. The Emory University Institutional Animal Care and Use Committee (IACUC) approved the mouse studies. Male and female *Sigmodon hispidus* cotton rats were bred and housed in the vivarium in Baylor College of Medicine. These cotton rats were ∼75 to 150 g of body weight at the start of the experiments, and all experimental protocols were approved by the Baylor College of Medicine's IACUC. Inbred *Sigmodon hispidus* cotton rats at Sigmovir Biosystems, Inc. (Rockville, MD) were utilized in a challenge study approved by the Sigmovir IACUC. All mouse and cotton rat experiments were conducted in accordance with the Guide for Care and Use of Laboratory Animals of the National Institutes of Health, as well as local, state and federal laws. Mice and cotton rats were randomly assigned to groups based on sequential selection from an inventory, and investigators were not blinded to outcome assessment.

### Assembly and rescue of recombinant RSV viruses

The following recombinant viruses were previously described: A2, A2-line19F, A2-line19F(M79I), A2-line19F(R191K), A2-line19F(K357T), A2-line19F(Y371N), A2-line19F(I557V), A2-line19F(K357T/Y371N) and A2-mKate2-2-20F/G^20^,^41^,^46^. The bacterial artificial chromosome (BAC) construct for OE4 was generated through modification of the published BAC containing A2-mKate2-line19F(I557V)^41^. The gene for monomeric Katushka 2 (mKate2, K), a far-red fluorescent reporter, is in the first gene position of the RSV antigenomic cDNA. Inclusion of mKate2 in this position did not attenuate RSV *in vitro* or in mice^41^. Deletion of SH (ΔSH) was performed by recombination-mediated mutagenesis (recombineering)^41^. The following oligonucleotides (Integrated DNA Technologies/IDT) were used to PCR-amplify the *galK* cassette such that the amplicon termini are homologous to the target site to replace SH with galK: dSH50f (5′-AGATCTAGTACTCAAATAAGTTAATAAAAAATATACACATGGACGTCCATCCTGTTGACAATTAATCATCGGCA-3′), where the underlined portions represent the 50 nt immediately upstream of the SH gene start in the BAC, and dSH50r (5′-GTCTTAGCGGTGCGTTGGTCCTTGTTTTTGGACATGTTTGCATTTGCCCCTCAGCACTGTCCTGCTCCTT-3′), where the underlined portion represents the complement of 50 nt beginning with the G gene start in the BAC. The non-underlined portions of the primers are specific to the *galK* cassette, as described^41^. Recombination in *E coli* resulted in replacing SH, from the beginning of the gene start to the end of the SH-G intergenic region, with the *galK* cassette. The following complementary oligonucleotides were annealed and used for removing the *galK* cassette in the second step of recombineering: dSH100f (5′-AGATCTAGTACTCAAATAAGTTAATAAAAAATATACACATGGACGTCCATGGGGCAAATGCAAACATGTCCAAAAACAAGGACCAACGCACCGCTAAGAC-3′) and dSH100r (5′-GTCTTAGCGGTGCGTTGGTCCTTGTTTTTGGACATGTTTGCATTTGCCCCATGGACGTCCATGTGTATATTTTTTATTAACTTATTTGAGTACTAGATCT-3′). Precise deletion of SH was confirmed by sequencing, yielding A2-K-ΔSH-line19F(I557V) BAC. The human codon-deoptimized NS1 (Supplementary Fig. 5) and NS2 (Supplementary Fig. 6) coding regions were digested from the BAC used for recovery of A2-dNSh previously described and ligated into the A2-K-ΔSH-line19F(I557V) BAC yielding an A2-K-dNSh-ΔSH-line19F(I557V)^8^. This construct was used for recovery of OE4+wild-type A2 G (termed OE4+wtG). Codon deoptimization of G was performed through substitution *in silico* of all codons least frequently used based on human codon usage bias into the RSV G sequence of A2. A point mutation (M48I) was introduced to ablate the secreted form of G (Supplementary Fig. 7). The coding region of codon-deoptimized G (dG) was synthesized by GenScript and cloned by restriction digestion and ligation into the A2-K-dNSh-ΔSH-line19F(I557V) BAC yielding A2-K-dNSh-ΔSH-dG-line19F(I557V) yielding the recovery BAC for OE4. The rescue of DB1 was previously described^9^. DB1–357/371 was generated through introduction of the line 19F residues K357 and Y371 into the DB1 coding sequence. The BAC for rescue of A2-del-M2-2 was generated by recombineering. We deleted 234 nt (from the seventh codon to the stop codon) of M2-2, as had been done previously for RSV ΔM2-2 (ref. 47). The following oligonucleotides were used to PRC-amplify the *galK* cassette for the first step of recombineering, delM2-1-f (5′-TTAGTGATACAAATGACCATGCCAAAAATAATGATACTACCTGACAAATACCTGTTGACAATTAATCATCGGCA-3′) and delM2-2-r (5′-ATTGTTTGAATTAATAATGTAACGATGTGGTGAGTGTTAGAATTGAGTGTTCAGCACTGTCCTGCTCCTT-3′). The following complementary oligonucleotides were annealed and used for the second recombineering step, M22_100f (5′-TTAGTGATACAAATGACCATGCCAAAAATAATGATACTACCTGACAAATAACACTCAATTCTAACACTCACCACATCGTTACATTATTAATTCAAACAAT-3′), and M22_100r (5′-ATTGTTTGAATTAATAATGTAACGATGTGGTGAGTGTTAGAATTGAGTGTTATTTGTCAGGTAGTATCATTATTTTTGGCATGGTCATTTGTATCACTAA-3′). Precise deletion of the targeted 234 nt was confirmed by sequencing. A version of OE4 without the mKate2 gene was also generated from pSynkRSV-dNS1-dNS2-ΔSH-line19F by excising the coding region containing the mKate2 gene with KpnI and AvrII. The resultant fragment containing mKate2 flanked by identical BlpI sites was then excised using BlpI, and the flanking fragments were ligated to generate pSynkRSV-dNS1-dNS2-ΔSH-line19F without mKate2. Recombinant viruses described in this paragraph were rescued in BSR-T7/5 cells^41^, and virus stocks were propagated in Vero cells.

The panel of RSV strains used for quantification of RSV nAb titres in cotton rat anti-sera were generated by first having cDNAs of F and G genes of the following A and B strains synthesized (GeneArt, Invitrogen): RSVA/1998/12-21 (JX069802), Riyadh A/91/2009 (JF714706/JF714710); and RSV B strains NH1276 (JQ680988/JQ736678), 9320 (AY353550), and TX11-56 (JQ680989JQ736679). The G and F gene segments were cloned into the A2-K BAC by restriction digestion and ligation, and the reporter viruses were recovered by transfection into BSR-T7/5 cells, followed by propagation of stocks in HEp-2 cells as was previously described^9^.

### RSV thermal stability

Virus aliquots were thawed at room temperature, pooled in 15 ml conical tubes, and mixed 1:1 with serum-free MEM before vortexing for 30 s. After vortexing, 1.5 ml of each virus suspension was transferred to replicate tubes to be incubated at either 4 or 37 °C in water baths. At designated time points, the tubes containing virus suspensions were removed, vortexed for 30 s each, and 300 μl was transferred to tubes then frozen in liquid nitrogen and stored at −80 °C. Quantification of titre was either determined by counting fluorescent focus units (FFU), if the virus was mKate2-expressing, or by plaque assay. For quantification by FFU titre, after all time points had been collected, samples were thawed on ice, vortexed 30 s, and serially diluted by 10-fold reductions in serum-free MEM in a 96 well plate. Once serially diluted, 50 μl of each dilution in triplicate was transferred to a 96-well plate containing confluent Vero cells. The virus was spinoculated onto the cells at 2,900*g* for 30 min at 4 °C before being overlaid with a 0.75% methylcellulose suspension in complete MEM. The plates were incubated at 37 °C for 2 days before FFU were counted. The methods for plaque assay have been previously described^20^. Plates of Vero cells infected for immunoplaque assay were incubated at 37 °C for 6 days prior to processing.

### Pre-F antigen ELISAs

Virus aliquots were thawed and diluted in MEM to yield high-titre stock suspensions. Then 100 μl of each virus stock suspension was added in triplicate to wells in a 96-well Costar Assay Plate, High Binding (Corning). The plates were covered and incubated at room temperature overnight. The next day, the virus suspension was dumped from the plate, and the plate was washed once with 150 μl per well of PBS-Tween (PBST, 0.05% Tween20 in PBS) followed by addition of 150 μl of 5% BSA (in PBS) per well for blocking. The plate was incubated at room temperature for 2 h. Pre-F-specific mAb MPE8 (ref. 19) was generated in HEK293-X2FreeStyle cells (U-Protein Express, BV) using human codon-optimized V_H_ and V_L_ sequences. Motavizumab mAb which binds pre-F and post-F was kindly provided by Nancy Ulbrandt (MedImmune/AZ). MPE8 and motavizumab antibodies were prepared by diluting the antibodies to 1 μg ml^−1^ in PBS before further dilution of 1:10,000 to 1:320,000 by serial dilutions in 1% BSA. Following blocking, the plate was washed once again with 150 μl per well of PBST before 100 μl of the serially diluted primary antibodies were applied to the wells. The plate was incubated for 2 h at room temperature before being dumped and washed three times with 150 μl per well of PBST. After washing, 100 μl of a 1:10,000 dilution of anti-human-HRP antibody in 1% BSA was applied and the plate incubated for 1 h at room temperature. Then the plate was dumped and washed three times with 150 μl of PBST before 100 μl of a pre-mixed reactive substrate reagent mixture (R&D Systems) was applied to catalyse a colorimetric reaction. The plate was covered and incubated for ∼10 min before the reaction was quenched by the addition of 100 μl of 0.2 N sulfuric acid. The plate was read at 450 nm on an ELISA plate reader. Background absorbance levels were subtracted from the test sample absorbance readings and plotted to a curve. The ratio of the area under the curve for MPE8 (pre-F) to the area under the curve for motavizumab (pre-F and post-F, total F) was calculated to determine pre-F level normalized to total F. The identical ELISA procedure was replicated using D25 instead of MPE8 as an additional measure of pre-F antigen levels.

To determine the stability of pre-F in OE4 compared with A2 at 4 °C over time, we incubated vials of virus for 0, 3 or 7 days and similarly applied 100 μl of each virus stock suspension in triplicate to wells in a 96-well Costar plate. We incubated the plates at 4 °C overnight, such that the final time points at time of measurement were 1, 4 and 8 days, respectively. We then performed ELISAs using MPE8 and motavizumab as above, but kept the plates and substrates at 4 °C or on ice for the remainder of the experiment.

### Western blotting

Western blots were performed on infected Vero cell lysates harvested in RIPA buffer as described^8^ using polyclonal rabbit antisera specific to NS1 (1:5,000) and NS2 (1:400; gifts from Michael Teng, USF Health), D14 (1:5,000; anti-RSV N; gift from Edward Walsh, University of Rochester), 131-2G (1:2,000; anti-RSV G, MAB858-2-5; Millipore), motavizumab (1:5,000; anti-F; gift from Nancy Ulbrant) or GAPDH (1:5,000), followed by peroxidase-conjugated anti-rabbit, anti-mouse or anti-human secondary antibodies (1:10,000; Jackson ImmunoResearch) (Fig. 3 and Supplementary Fig. 8). Densitometry analyses were performed using Image Lab software (Bio-Rad).

### Viral replication in immortalized and primary cell cultures

The media from 70% confluent Vero or BEAS-2B cells in six-well plates was aspirated, and 0.5 ml of virus at a multiplicity of infection (MOI) of 0.01 was added to replicate wells for each of the time points to be acquired for each virus strain. The plates were rocked at room temperature for 1 h. Following infection, the virus was carefully aspirated and the monolayers washed twice with 1 ml of PBS before 2 ml of pre-warmed complete E-MEM (Vero) or RPMI (BEAS-2B) was added. The plates were incubated at 37 °C and 5% CO_2_ for the duration of the time courses. Time points were acquired at 1, 12, 24, 36, 48, 72 and 96 h post infection. At each time point, the monolayers were scrapped into the supernatant, vortexed briefly and flash frozen in liquid nitrogen before storage at −80 °C. NHBE cells from two donors were differentiated at ALI and the monolayers washed with PBS before being infected apically with 100 μl of virus at an MOI of 2.6. The virus was left to incubate for 2 h at 37 °C before removal and three subsequent washes with PBS. At designated time points, 150 μl of differentiated medium without inducer was incubated on the apical surface for 10 min at 37 °C before harvesting and transfer into microcentrifuge tubes. The process was repeated to yield a total of 300 μl of pooled apical wash, which was frozen in liquid nitrogen and stored at −80 °C for later titration. Similar to the NHBE infection, HAE cells from two donors were differentiated at ALI, the apical surface washed with PBS, and infected with an initial MOI of 6.7. Following 2 h incubation at 37 °C, the virus inoculum was aspirated, the apical layer washed three times with PBS and the culture incubated at 37 °C. For each designated time point, the apical layers were washed with 425 μl of media for 30 min at 37 °C and the supernatant stored at −80 °C. FFU titration was performed for all analyses as described above on either HEp-2 or Vero cells.

### Attenuation and efficacy in mice

For determination of viral load, 7-week-old female BALB/c mice (Charles River) were infected i.n. under sedation with 100 μl of virus in serum-free MEM. On days 2, 4, 6 and 8, the mice were euthanized and the left lung harvested and homogenized for viral FFU titre assay. Titres below the limit of detection were assigned a value equal to half of the limit of detection. To assess the genetic stability of OE4 NS1, NS2, F and G genes *in vivo*, we also used the lung homogenate from day 6 post infection to sequence the genes of interest after passage in mice. We first isolated viral RNA directly from lung homogenate using Nucleospin RNA purification kit (Macherey-Nagel) and performed reverse transcription using SuperScript III reverse transcriptase (Thermo-Fisher). We then amplified regions of interest using PfuTurbo DNA polymerase (Agilent) and obtained sequences via GenHunter Corp.

For determination of serum nAb titres and challenge studies, 7-week-old female BALB/c mice (Jackson) were infected i.n. with 100 μl of virus in serum-free MEM. On days 35, 70 and 100, the mice were sedated and serum samples obtained via submandibular vein bleeding. Sera were stored at −80 °C until quantification by a FFU microneutralization assay^8^. Neutralization titres were determined by co-incubating heat-inactivated (56 °C, 30 min) sera, which had been two-fold serially diluted with 50–100 FFU of virus for 1 h at 37 °C. The serum-virus mixtures were then spinoculated onto HEp-2 monolayers in 96-well plates at 2,900*g* for 30 min at 4 °C before being overlaid with 0.75% methylcellulose in complete MEM. FFU per well were counted 2 days later, and EC50 titres were determined by nonlinear regression analysis (GraphPad Prism). To challenge the mice after vaccination, the mice were sedated on day 102 post inoculation and infected i.n. with 10^5^ PFU A2-line19F. After 4 days, the viral load was determined on the left lung by plaque assay on HEp-2 cells.

### Histopathology in mice

Female 8-week-old BALB/c mice (Jackson) were sedated and infected intranasally with either mock solution or 10^5^ FFU of A2, A2-line19F, OE4 or A2-del-M2-2. After 8 days, the lungs were harvested, fixed, sectioned and stained with Periodic acid-Schiff (PAS). Morphometric quantification of airway PAS positivity was performed on digitized slides using a Mirax digital pathology system (Zeiss) and Histoquant software as previously described^20^. All airways in the sections were analysed.

### Attenuation and efficacy in cotton rats

For determination of viral load in naive animals, 8- to 10-week-old male and female cotton rats were sedated and inoculated i.n. with 10^5^ FFU of virus in 100 μl in serum-free MEM at Baylor College of Medicine. On day 4 post infection, the cotton rats were killed. For acquisition of lung lavage washes, the left lobe of the lung was excised and transpleurally lavaged with 3 ml of Iscove's media with 15% glycerin mixed with 2% FBS-MEM (1:1). For acquisition of nasal wash, the jaws were first disarticulated and the head was removed. A solution of 1 ml of Iscove's media with 15% glycerin mixed with 2% FBS-MEM (1:1) was washed through each nare for a total of 2 ml of volume. Titration was performed by plaque assay on HEp-2 cell monolayers. For determination of nAb titres, 8- to 10-week-old male and female cotton rats at Baylor College of Medicine were sedated and inoculated i.n. with 10^5^ FFU of virus in 100 μl of serum-free MEM. On day 42, serum was obtained via the orbital plexus of the cotton rats and stored at −80 °C until analysed as described above. To assess efficacy, cotton rats were challenged on day 42 post infection (following i.n. vaccination with 3 × 10^5^ FFU) with 1 × 10^6^ FFU i.n. of RSV strain A2-line19F at Sigmovir Biosystems Inc. On day 4 post challenge, the nasal turbinates were homogenized in 3 ml of HBSS supplemented with 10% SPG, and the left lung was homogenized in 3 ml of HBSS supplemented with 10% SPG. The nasal and lung tissue titres were determined by plaque assay as described above.

### Enhanced disease study in cotton rats

Groups of five 6- to 8-week-old female cotton rats were vaccinated intranasally with either 10^5^ FFU of OE4, MEM (mock treatment), or intramuscularly with FI-RSV (lot 100; 1:125) at Sigmovir, Inc. On day 21, FI-RSV-vaccinated rats were boosted with a second identical vaccination. On day 42, all cotton rats were challenged intranasally with 1.35 × 10^5^ PFU of A2-line19F. On day 48 (day 6 post challenge), the cotton rats were killed and the lungs excised, perfused with 10% formalin and sections of paraffin-embedded inflated lungs were stained with hematoxylin-eosin (H&E). The slides were scored by a pathologist blinded to the groups on a scale of 0 to 4 based on increasing severity of peribronchial mononuclear inflammatory cell infiltration, perivascular mononuclear inflammatory cell infiltration, interstitial pneumonitis and alveolitis.

### Cryo-ET

BEAS-2B cells were seeded on gold R2/1 Quantifoil TEM grids in MatTek dishes and were infected when subconfluent (30–40%) at an MOI of 10 using A2 and OE4 strains. Twenty-four hours post infection, infected cells on gold Quantifoil TEM grids were plunge frozen using a Gatan CryoPlunge 3 apparatus (Gatan, Inc., Pleasanton, CA). For the A2 strain that was heat-treated, BEAS-2B cells were seeded on gold R2/1 Quantifoil TEM grids in MatTek dishes and were infected when subconfluent (30–40%) at an MOI of 10 with the A2 strain. Twenty-four hours post infection, the MatTek dishes containing TEM grids were incubated for 30 min at 55 °C (ref. 23). Immediately after heat treatment, infected cells on gold Quantifoil TEM grids were plunge frozen using a CryoPlunge 3 apparatus. In all instances, 4 μl of BSA-conjugated 10 nm gold nanoparticles was applied onto the TEM grid prior to cryoplunging. Cryogrids were stored in liquid nitrogen prior to imaging with a JEOL JEM-2200FS TEM at 200 kV (JEOL Ltd., Japan), which is equipped with a field emission gun, an in-column Omega energy filter with a slit width of 20 eV. Tilt series were recorded semi-automatically using the SerialEM package from −65° to 65° at 2° increment step, −6 μm defocus, with a total dose of ∼120 e^−^ Å^−2^ (refs 17, 38, 48, 49). Images were recorded on a Direct Electron DE-20 camera (Direct Electron, LP, San Diego, CA) at 12 frames per second at a nominal magnification of 10,000 resulting in a pixel size of 0.614 nm.

Tilt series frames were motion corrected prior to tomographic reconstruction using python scripts provided by the manufacturer (Direct Electron, LP). Motion corrected frames were used for tomographic reconstruction in the IMOD software package using the weighted back-projection algorithm, and the 10 nm gold nanoparticles were used as fiducials to align frames at the different tilt angles^50^. Reconstructed three-dimensional volumes (unbinned and binned by a factor of 2) were also CTF-corrected by inversing the phase and de-noised by nonlinear anisotropic diffusion.

### Subvolume averaging and model fitting

Subvolumes of RSV glycoproteins were manually selected (3,827, 2,567 and 1,313 subvolumes for A2, OE4 and A2-heat, respectively) from tomograms binned by a factor of 2, using EMAN2 *e2spt_boxer.py* script^51^. Initially, two-fold binned data were used in the subvolume averaging process. Alignments and averaging were performed in PEET 1.11.0 Alpha version, and each subvolume was normalized (‘flgNormalize=1') prior to alignment and averaging^52^. Initial orientations of the subvolumes were determined using SpikeInit. Particles were considered duplicates if the centre-to-centre distance was <60 Å for A2 and OE4 and 40 Å for A2-heat samples; only the ones with the highest cross-correlation coefficient values were kept. The initial reference was a previously published post-fusion F glycoprotein (EMDB-2393) low-pass filtered to 60 Å. A soft-edged cylinder mask was applied during alignment to eliminate contributions from the neighbouring particles. Using the two-fold binned data, six iterations were run with missing wedge compensation (eight weight groups) and the resulting averages indicated three-fold symmetry, consistent with the crystal structures. Thus, we imposed C3 symmetry by creating a three-fold symmetric data set: the first set are the aligned particles, the second and third sets have all the same tilt angles and positions as the first set, but with either 120° or 240° of twist rotation along the *y* axis applied using *modifyMotiveList* in PEET. The initial subvolume averages were used as references for refinements with C3 symmetry imposed, and three more iterative refinements were run with smaller transitional and angular search ranges and increasing high-frequency cutoff values. The respective translation information from the two-fold binned data were scaled by a factor of two to match the unbinned tomograms, and were used as input MotiveList for three more iterative refinements on the unbinned data. The final subvolume averages (final pixel size of 6.14 Å, unbinned) with C3 symmetry were reconstructed from 2,268, 1,687 and 823 subvolumes, for A2, OE4 and A2-heat, respectively. The final density maps of F were low-pass filtered to FSC=0.143 cutoff calculated in PEET, and masked using a soft edged cylinder generated using SPIDER^53^. The atomic crystal structures of pre-fusion and post-fusion F glycoprotein (PDB IDs 4JHW and 3RRT, respectively) were manually fitted into the final electron density maps using Chimera^54^.

### Immuno-TEM

BEAS-2B cells were seeded on Alcar disks in 24-well plates and were infected when subconfluent (50–70%) at an MOI of 10 using A2 and OE4 strains. Twenty-four hours post infection, anti-pre-F (MPE8)^19^, anti-post-F (131-2A)^33^,^55^, anti-F (motavizumab, gift from Nancy Ulbrant) and anti-G (131-2G, MAB858-2-5; Millipore), primary antibodies were added to RPMI-1640 medium at a final concentration of 5 μg ml^−1^. After primary antibody incubation for 1.5 h at 37 °C, cells were washed four times with RPMI-1640 medium, and then incubated for 1.5 h at 37 °C with goat anti-human (used for MPE8 and motavizumab) or goat anti-mouse (used for 131-2A and 131-2G) secondary antibody conjugated to 6 nm gold particles in RPMI-1640 medium at a final concentration of 10–20 μg ml^−1^. Following additional medium washes, cells were fixed in 2.5% glutaraldehyde at 4 °C overnight. The next day, fixed cells on Aclar disks were washed with 0.1 M phosphate butter (pH 7.4) followed by pre-fixation with 1% OsO_4_ in 0.1 M phosphate buffer for 1 h. The cells were then washed with deionized water before dehydration at 5 min intervals in graded concentrations of ethanol (25, 50, 75, 95 and 100%). The cells were then treated with a 1:1 resin mixture of 100% ethanol and Eponate 12 for 1 h, followed by polymerization with 100% Eponate 12 resin overnight in the oven. Ultrathin sections were cut between 60 and 80 nm in thickness, and then stained using 5% uranyl acetate and 2% lead citrate. Sections were imaged as montages using SerialEM software on a JEOL JEM-1400 TEM (JEOL Ltd., Japan) equipped with a Gatan US1000 2 k × 2 k CCD camera (Gatan) at × 8,000 nominal magnification^17^,^18^,^56^.

Montages were assembled using sloppyblend.com script, and further measurements and quantification were done using the blended maps. The six groups of data (70 montages, 1,515 viral particles) were randomly blinded among groups prior to quantification. Total membrane length of a viral particle was measured using *imodinfo* command by placing open model points along the viral membrane, where distinct membrane morphology is present. The 6 nm immunogold particles on both sides of viral particle membranes were counted separately. Gold particle intensity per particle was calculated using total gold particles on the viral membrane divided by total viral membrane length (gold particles per μm of membrane length). Representative images for the immuno-TEM were selected based on the average gold particle intensities along the membrane.

### Statistical analyses

All statistical analyses were computationally performed using GraphPad Prism. The number of replicates and type of statistical analysis performed are described for all experiments in the figure legends. No statistical methods were used in predetermining sample sizes.

### Data availability

All relevant data are available from the authors upon request.

## Additional information

**How to cite this article:** Stobart, C. C. *et al*. A live RSV vaccine with engineered thermostability is immunogenic in cotton rats despite high attenuation. *Nat. Commun.* **7,** 13916 doi: 10.1038/ncomms13916 (2016).

**Publisher's note:** Springer Nature remains neutral with regard to jurisdictional claims in published maps and institutional affiliations.

## Supplementary Material

Supplementary Information Supplementary Figures.
