# Supplementary material for: A live RSV vaccine with engineered thermostability is immunogenic in cotton rats despite high attenuation
Source: Nat Commun. 2016 Dec 21;7:13916. doi: 10.1038/ncomms13916 (PMC5187593; doi:10.1038/ncomms13916)
Supplement: Supplementary Information — Supplementary Figures. [file ncomms13916-s1.pdf]

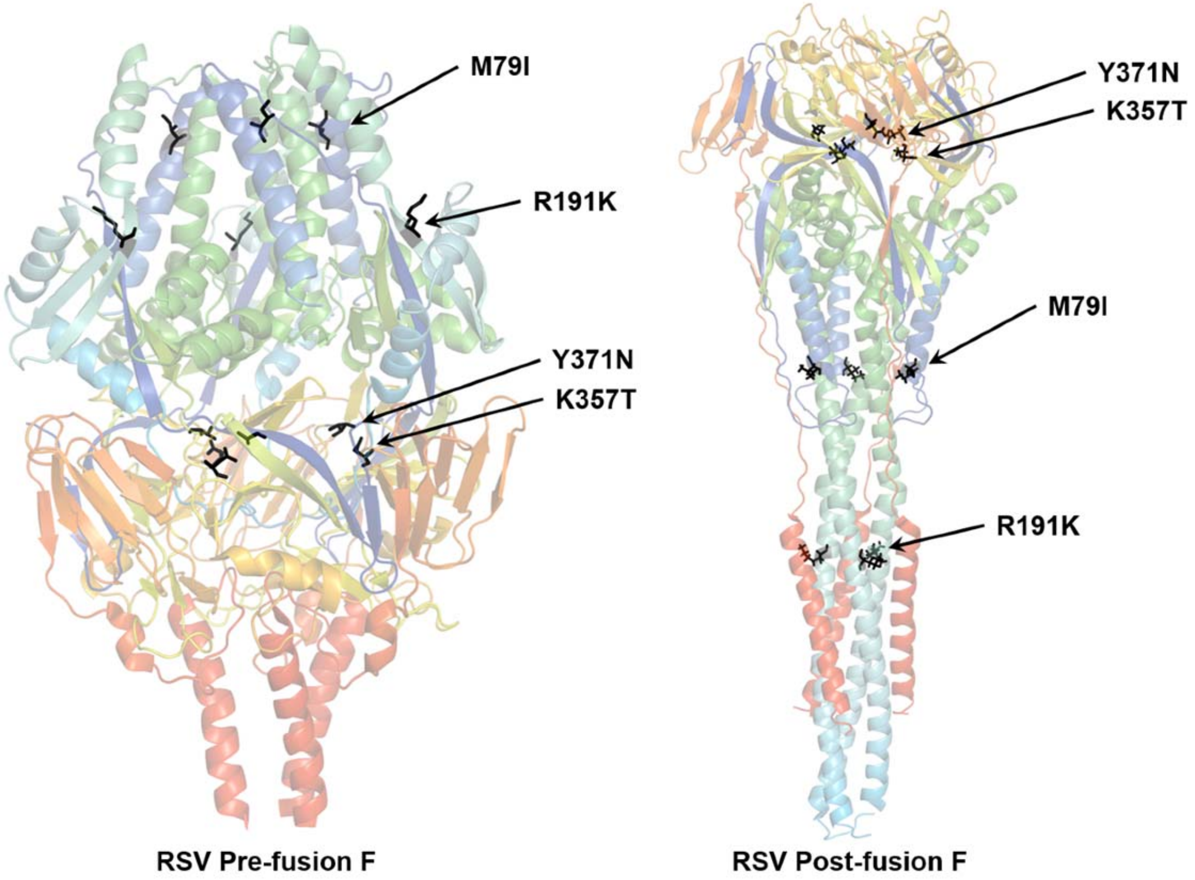

Supplementary Figure 1. Pre-fusion (pre-F) and post-fusion F (post-F) conformations labeled with four unique residues of line 19 F. The labeled images of pre-F (PDB accession number 4JHW) and post-F (PDB accession number 3RRT) were generated using previously described crystal structures[10].

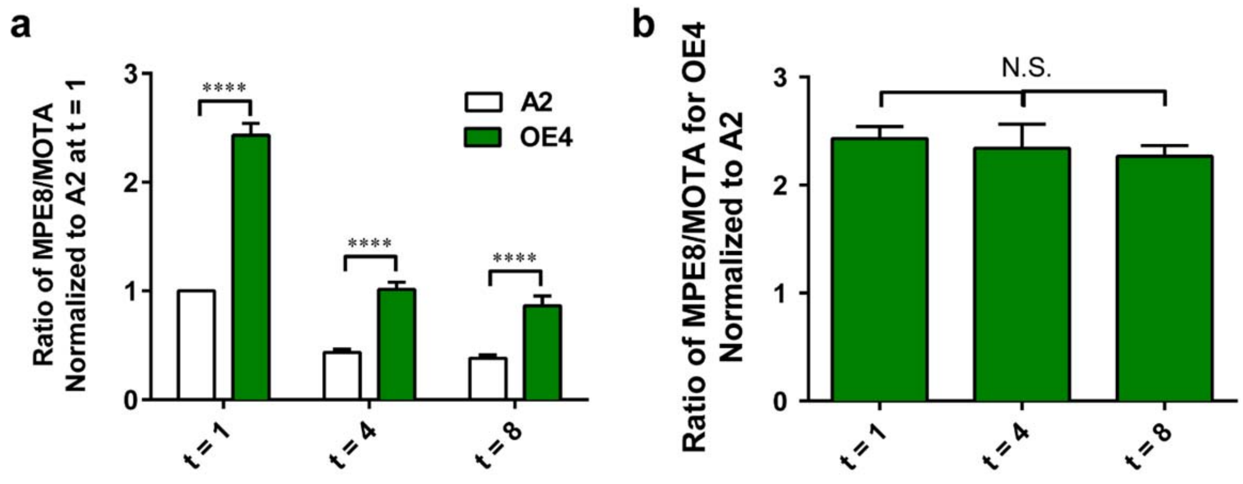

Supplementary Figure 2. Stability of A2 and OE4 pre-F over time at 4°C. (**a**) Ratio of direct ELISA using MPE8, a pre-F specific mAb, to direct ELISA using motavizumab, a total F mAb., on virus incubated at 4°C over 8 days. Ratio values are shown without normalization. (**b**) Normalized ratios of OE4 to A2 at days 1, 4, and 8. t = number of days at 4°C. All data for each time point reflect the means + standard deviations of triplicate series of serial dilutions of virus after incubation. Statistical analyses were performed by one-way ANOVA, (****, $P < 0.00005$).

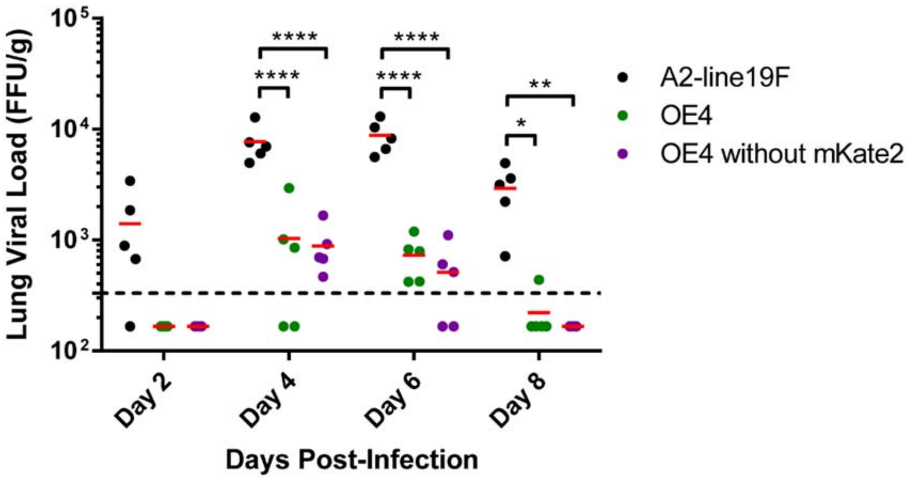

Supplementary Figure 3. Comparison of mouse lung viral loads of OE4 with and without mKatushka-2 reporter. BALB/c mice (n=5) were infected i.n. with 4 x 10^5 PFU of virus per mouse. On days 2, 4, 6, and 8, the mice were euthanized and the left lung harvested for viral titration. Titers below the limit of detection were assigned a value equal to half of the limit of detection (dashed line). Means are indicated by red bars. Statistical analyses were performed by two-way ANOVA, (*, $P < 0.05$; **, $P < 0.005$; ***, $P < 0.0005$); ****, $P < 0.00005$).

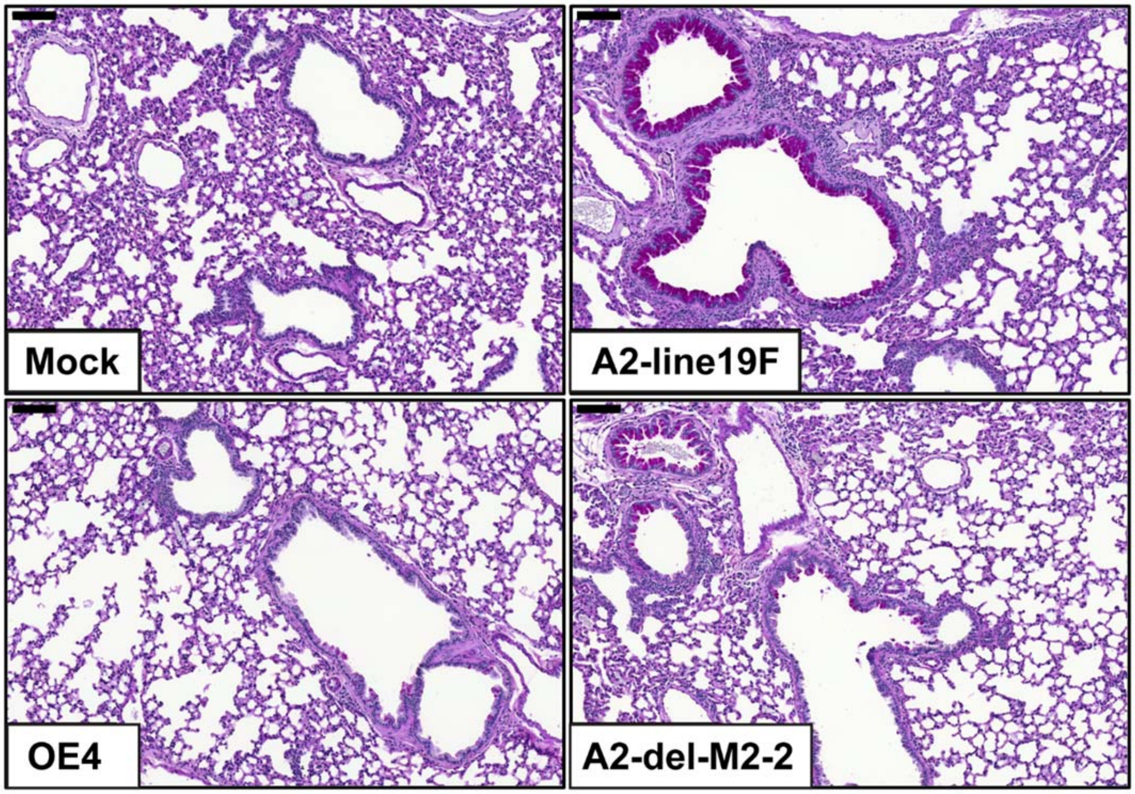

Supplementary Figure 4. Representative airway mucin expression. Mice (5 per group) were inoculated with mock, A2-line19F, OE4, or A2-del-M2-2, and lungs were harvested 8 days post-inoculation for sectioning and staining with periodic acid Schiff (PAS). Representative images are shown. Scale bars are 100 μm.

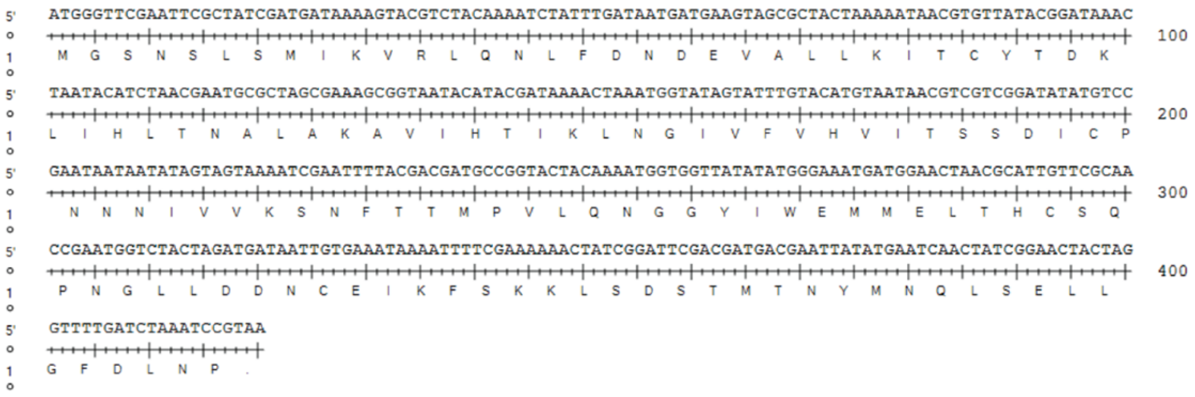

Supplementary Figure 5. Nucleotide and amino acid sequences of codon-deoptimized non-structural protein NS1.

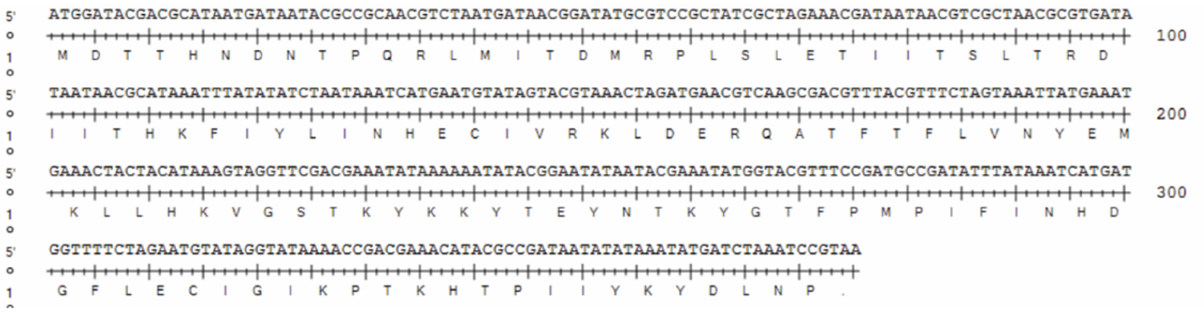

Supplementary Figure 6. Nucleotide and amino acid sequences of codon-deoptimized non-structural protein NS2.

```
5' ATGTCGAAAAATAAAGATCAACGTACGGCGAAAACGCTAGAACGTACGTGGGATACGCTAAATCATCTACTATTTATATC 80
1   M  S  K  N  K  D  Q  R  T  A  K  T  L  E  R  T  W  D  T  L  N  H  L  L  F  I  S

5' GTCGTGTCTATATAAACTAAATCTAAAATCGGTAGCGCAAATAACGCTATCGATACTAGCGATAATAATATCGACGTCGC 160
1    S  C  L  Y  K  L  N  L  K  S  V  A  Q  I  T  L  S  I  L  A  I  I  I  S  T  S

5' TAATAATAGCGGCGATAATATTTATAGCGTCGGCGAATCATAAAGTAACGCCGACGACGGCGATAATACAAGATGCGACG 240
1  L  I  I  A  A  I  I  F  I  A  S  A  N  H  K  V  T  P  T  T  A  I  I  Q  D  A  T

5' TCGCAAATAAAAAATACGACGCCGACGTATCTAACGCAAAATCCGCAACTAGGTATATCGCCGTCGAATCCGTCGGAAAT 320
1   S  Q  I  K  N  T  T  P  T  Y  L  T  Q  N  P  Q  L  G  I  S  P  S  N  P  S  E  I

5' AACGTCGCAAATAACGACGATACTAGCGTCGACGACGCCGGGTGTAAAATCGACGCTACAATCGACGACGGTAAAAACGA 400
1    T  S  Q  I  T  T  I  L  A  S  T  T  P  G  V  K  S  T  L  Q  S  T  T  V  K  T

5' AAAATACGACGACGACGCAAACGCAACCGTCGAAACCGACGACGAAACAACGTCAAAATAAACCGCCGTCGAAACCGAAT 480
1  K  N  T  T  T  T  Q  T  Q  P  S  K  P  T  T  K  Q  R  Q  N  K  P  P  S  K  P  N

5' AATGATTTTCATTTTGAAGTATTTAATTTTGTACCGTGTTCGATATGTTCGAATAATCCGACGTGTTGGGCGATATGTAA 560
1   N  D  F  H  F  E  V  F  N  F  V  P  C  S  I  C  S  N  N  P  T  C  W  A  I  C  K

5' ACGTATACCGAATAAAAAACCGGGTAAAAAAACGACGACGAAACCGACGAAAAAACCGACGCTAAAAACGACGAAAAAAG 640
1    R  I  P  N  K  K  P  G  K  K  T  T  T  K  P  T  K  K  P  T  L  K  T  T  K  K

5' ATCCGAAACCGCAAACGACGAAATCGAAAGAAGTACCGACGACGAAACCGACGGAAGAACCGACGATAAATACGACGAAA 720
1  D  P  K  P  Q  T  T  K  S  K  E  V  P  T  T  K  P  T  E  E  P  T  I  N  T  T  K

5' ACGAATATAATAACGACGCTACTAACGTCGAATACGACGGGTAATCCGGAACTAACGTCGCAAATGGAAACGTTTCATTC 800
1   T  N  I  I  T  T  L  L  T  S  N  T  T  G  N  P  E  L  T  S  Q  M  E  T  F  H  S

5' GACGTCGTCGGAAGGTAATCCGTCGCCGTCGCAAGTATCGACGACGTCGGAATATCCGTCGCAACCGTCGTCGCCGCCGA 880
1    T  S  S  E  G  N  P  S  P  S  Q  V  S  T  T  S  E  Y  P  S  Q  P  S  S  P  P

5' ATACGCCGCGTCAAtag
1  N  T  P  R  Q  .
```

Supplementary Figure 7. Nucleotide and amino acid sequences for codon-deoptimized attachment G protein.

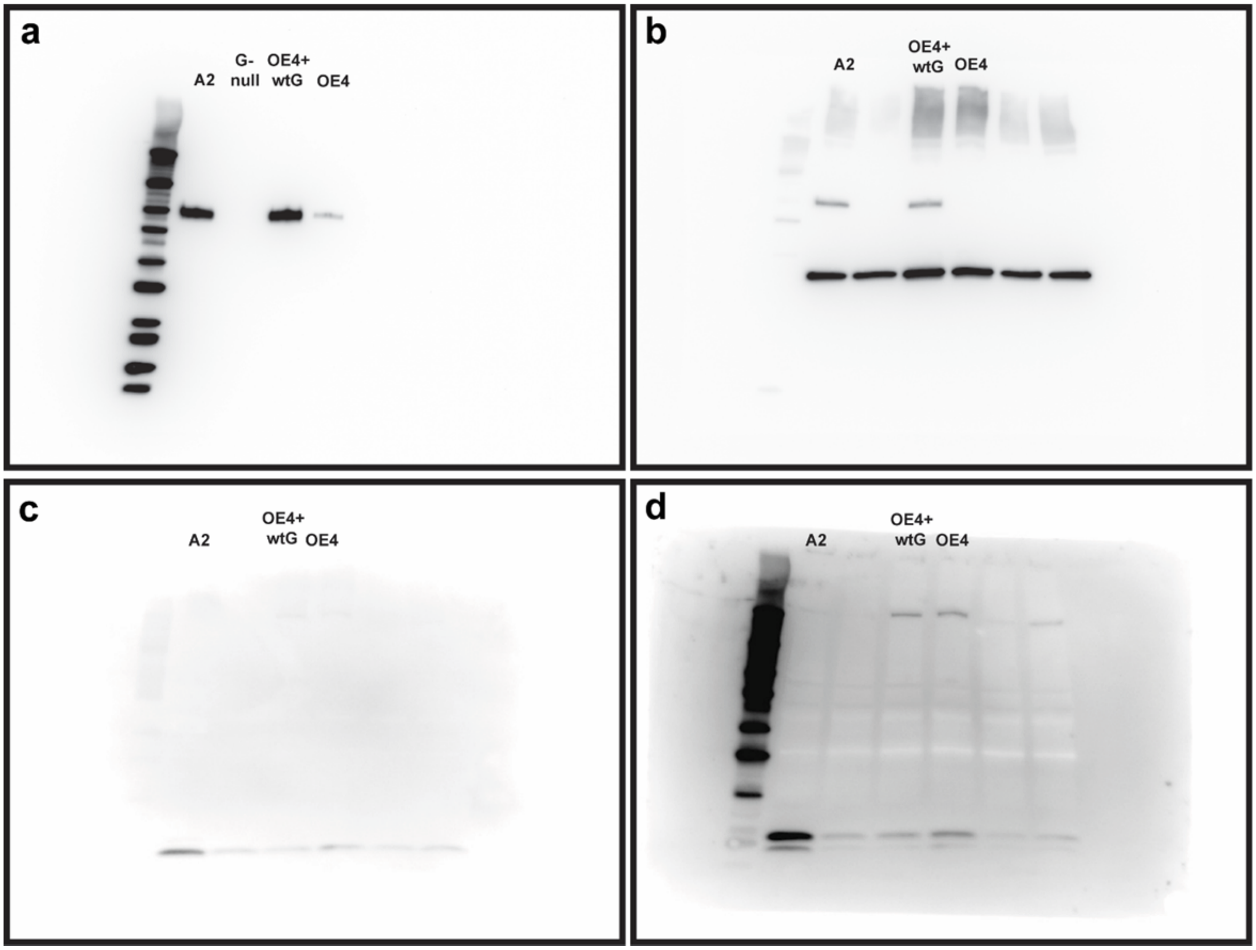

Supplementary Figure 8. Raw western blot images for Figure 3b. Western blotting of Vero cells infected with A2, A2-G-null, OE4 expressing wild-type G (OE4-wtG), or OE4 were performed for G (a), N (b), NS1 (c), and NS2 (d).
